# Optimization of culture conditions to obtain maximal growth of penicillin-resistant *Streptococcus pneumoniae*

**DOI:** 10.1186/1471-2180-5-34

**Published:** 2005-06-02

**Authors:** Andrea V Restrepo, Beatriz E Salazar, María Agudelo, Carlos A Rodriguez, Andres F Zuluaga, Omar Vesga

**Affiliations:** 1 GRIPE: Grupo Investigador de Problemas en Enfermedades Infecciosas, University of Antioquia Medical School, Medellín, Colombia; 2 Section of Infectious Diseases, Department of Internal Medicine, University of Antioquia Medical School, Medellín, Colombia; 3 Department of Pharmacology & Toxicology, University of Antioquia Medical School, Medellín, Colombia

## Abstract

**Background:**

*Streptococcus pneumoniae*, particularly penicillin-resistant strains (PRSP), constitute one of the most important causes of serious infections worldwide. It is a fastidious microorganism with exquisite nutritional and environmental requirements to grow, a characteristic that prevents the development of useful animal models to study the biology of the microorganism. This study was designed to determine optimal conditions for culture and growth of PRSP.

**Results:**

We developed a simple and reproducible method for culture of diverse strains of PRSP representing several invasive serotypes of clinical and epidemiological importance in Colombia. Application of this 3-step culture protocol consistently produced more than 9 log_10 _CFU/ml of viable cells in the middle part of the logarithmic phase of their growth curve.

**Conclusion:**

A controlled inoculum size grown in 3 successive steps in supplemented agar and broth under 5% CO_2 _atmosphere, with pH adjustment and specific incubation times, allowed production of great numbers of PRSP without untimely activation of autolysis mechanisms.

## Background

*Streptococcus pneumoniae*, one of the most important human pathogens of all times, is today an even more serious menace because of its increasing resistance to penicillin, the first-choice antibiotic [1]. It is a fastidious organism to grow both in vitro and in animal infection models. Probably because of the wide genetic diversity of this organism and its exclusive nature as human pathogen, it is difficult to obtain comparable cell production when culturing different strains even within the same serotype [2].

In the field of experimental animal models there is also wide variation in the susceptibility of mice strains and specific organs to infection with *S. pneumoniae *[3,4]. While new drugs with activity against penicillin-resistant *S. pneumoniae *(PRSP) are being discovered, only penicillin-susceptible strains grow in the lungs of experimental animals with some consistency [5]. Although other organs such as the thighs of neutropenic mice are susceptible to progressive infection with PRSP, a reproducible pneumonia model remains elusive for most strains of PRSP.

Little attention has been given to the culture conditions of *S. pneumoniae*, critical to obtain the appropriate amount of viable log-phased cells to inoculate the animals. *S. pneumoniae *was reclassified within the group of anaerobic bacteria because its relatively small genome simply lacks many genes required for aerobic growth [6]. As a Gram-positive catalase-negative coccus that generates H_2_O_2 _via a flavoenzime system, it grows better in presence of a source of catalase such as red blood cells [7,8]. In contrast to most bacteria, *S. pneumoniae *also requires choline for growth in defined media, and reducing agents are almost essential. Growth of most strains requires vitamin B complex (biotin, nicotinamide, pantothenate, pyridoxal, riboflavin and thiamine), adenine, guanine, uracil, and 7–10 amino acids. Its energy metabolism is fermentative, yielding primarily low levels of lactic acid, but optimum pH for growth is 7.8 with a range of 6.5–8.3 [9]. Besides these limitations, *S. pneumoniae *displays a particularly effective quorum sensing system that activates several potent autolysins once certain conditions are met within the growing population, and this is perhaps the most important hurdle to overcome when trying to obtain viable log-phased cells [10]. Among several factors not well understood, culture acidification is one of the conditions met by the growing population that clearly contributes to start the autolysis process [11].

We hypothesized that optimization of culture conditions tending to prevent environmental acidification and its consequent activation of autolysis mechanisms could lead to better production of PRSP in vitro, and aimed to determine and provide such ideal conditions for the growth of this microorganism, so they could be applied to diverse strains of clinical importance to develop a reproducible animal model of pneumonia.

## Results

### Susceptibility testing

Minimal inhibitory (MIC) and bactericidal (MBC) concentrations of six antimicrobial agents for these strains are shown in Table 1. Reduced susceptibility to penicillin was demonstrated for 6 strains, the other 2 were susceptible. Among those non-susceptible, 4 strains displayed intermediate (MIC 0.12–1 μg/mL) and 2 full resistance to penicillin (MIC > 1 μg/mL). Only one strain (INS-E685) was susceptible to all six antibiotics tested, but all of them were susceptible to erythromycin and vancomycin. MIC of ceftriaxone against PRSP INS-E611, E674 and E676 was 1 μg/mL, a value conferring intermediate resistance for CSF isolates, as was the case for strain INS-E676 (Table 1).

### Evaluation of baseline culture variables

The results are summarized in Table 2. For cryoprotection, skim milk invariably allowed recuperation of frozen organisms, while 17% glycerol failed once (no viable bacteria after thawing). During Phase 0, we found that two successive passes on solid media were necessary for complete recuperation after thawing, and that their optimal incubation time was 15 hours. After 15 hours colony umbilication became deeper, suggesting progression of the autolysis process [9]. The number of colonies from Phase 0 employed to inoculate Tube 1 in Phase 1 was critical to maximize the number of viable log-phased cells: 10 colonies into 10 ml of culture broth gave the best results. Since broth acidification is known to start autolysis, a low pH after 12 hours of incubation during Phase 1 was used as an indicator of poor viability. Compared with 10 colonies, 5 or less produced lower cell numbers with similar decrease in pH, and 15 or more colonies gave greater cell numbers but with a profound decrease in pH (Table 3). Besides 5% sheep blood, additional supplementation of trypticase soy agar (TSA) with 0.5% yeast extract (instead of 0%) during Phase 0 increased the number of viable cells produced by broth culture in Phase 1 from 6.60–7.60 to 8.32–8.63 CFU/ml.

**Figure 1:**
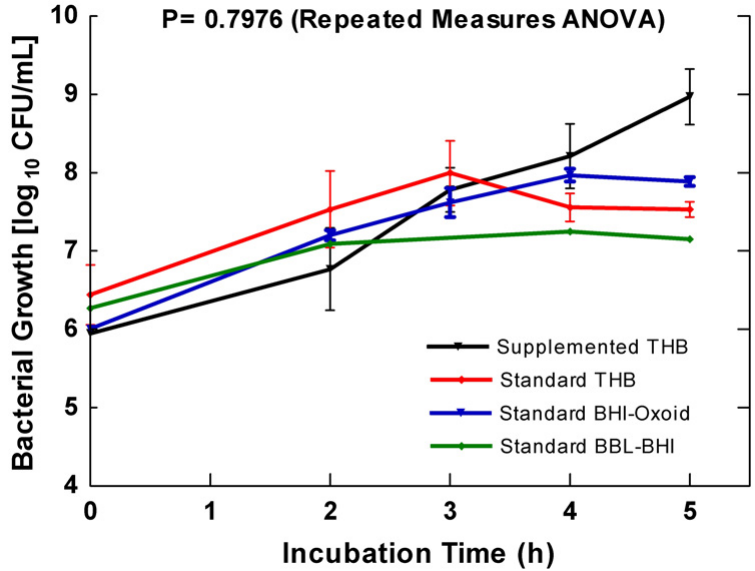
**Comparison of three standard liquid media with a supplemented medium for culture of *S. pneumoniae *INS-E611**. Five-hour growth curves for a PRSP strain in four different culture media: standard BHI (two brands), standard THB, and supplemented THB. All curves were obtained from Tube 7 (Phase 2). Bacterial growth was poor in standard BBL-BHI, and was not used for further experiments. As expected, there was no difference among these media when every data point along time was compared by repeated measures ANOVA (P = 0.7976). However, there was a significant difference in maximal growth between supplemented THB and standard media, as illustrated by Figure 2.

**Figure 2:**
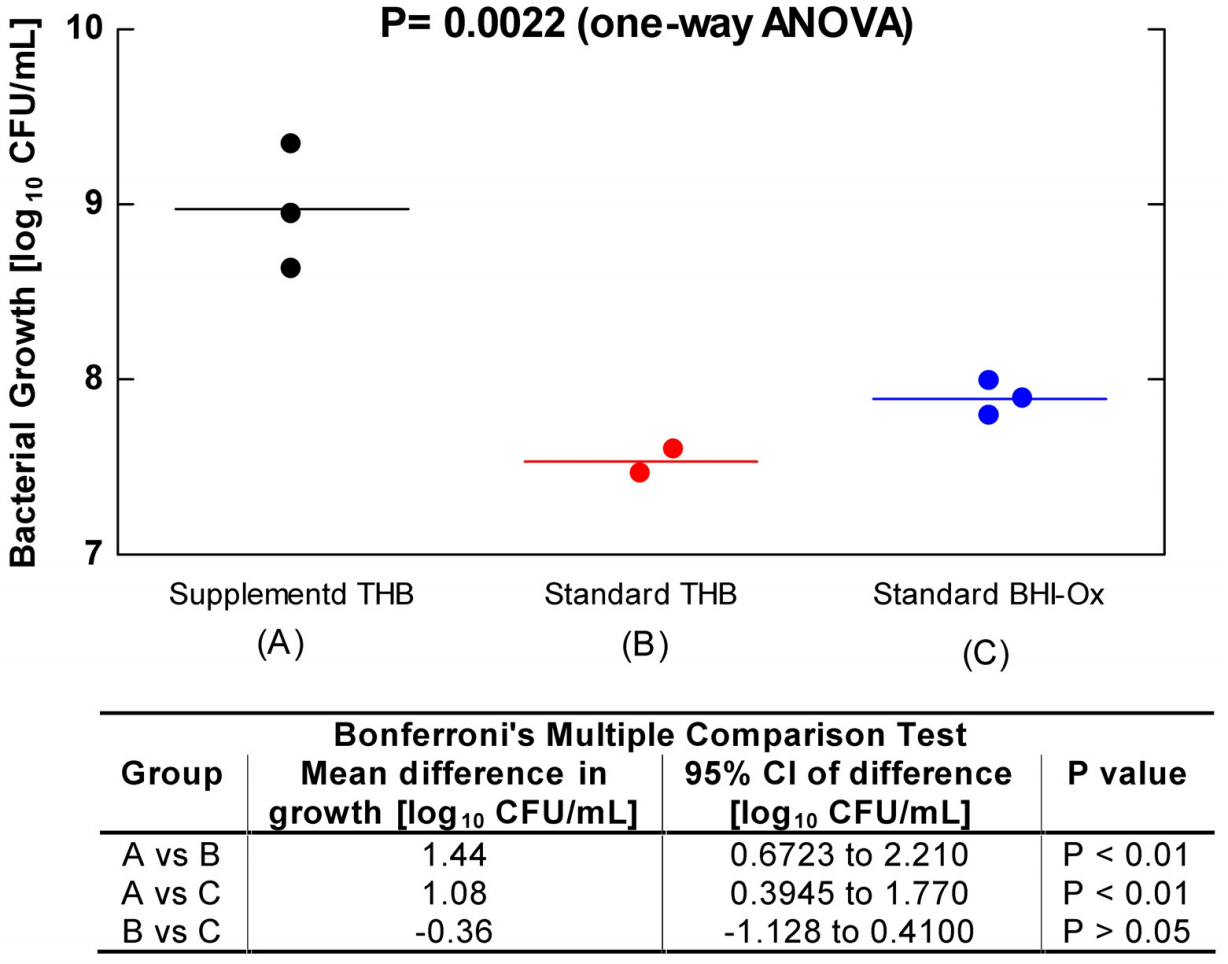
**Maximal growth of *S. pneumoniae *INS-E611 cultured in three liquid media**. The graph illustrates cell production of each medium after 5 hours of incubation; all cultures were obtained from Tube 7 (Phase 2). Supplemented THB was clearly superior to standard THB and BHI.

**Figure 3:**
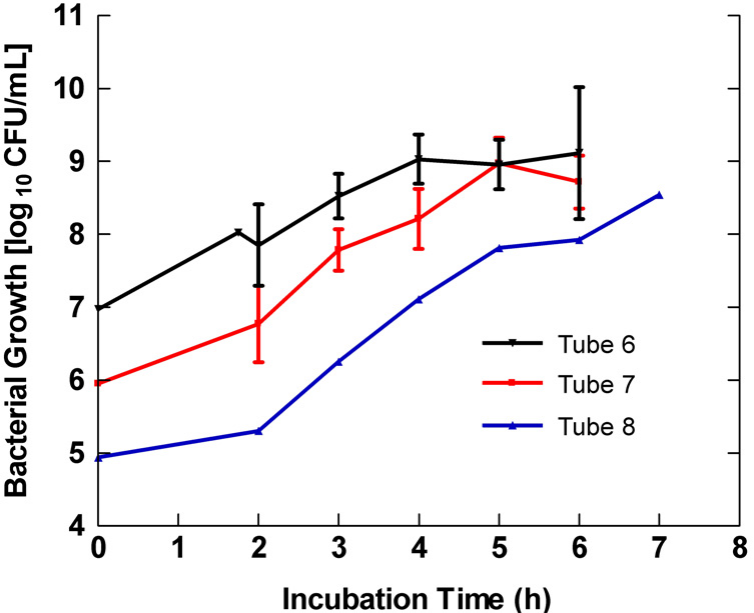
**Growth of *S. pneumoniae *INS-E611 during Phase 2 in supplemented THB**. One-mL bacterial suspension produced by Tube 5 (Phase 1) after 12 h of incubation was inoculated into 9-mL fresh broth (Tube 6, 1:10 dilution) and diluted further into Tube 7 (1:100) and Tube 8 (1:1000). Incubation under 5% CO_2 _atmosphere and pH adjustment every hour followed for 5–6 hours, with simultaneous plating for CFU counting. The graph illustrates the time to reach maximum growth for each inoculum size before triggering autolysis: 4, 5 and 7 hours for Tubes 6, 7 and 8, respectively. With this method, very similar maximum growths were attained under different inoculum sizes. Experiments with each tube were done 1–3 times.

The type of liquid media was tested before (standard broth) and after diverse adjustments (supplemented broth). Standard Todd Hewitt Broth (THB) was more reliable than Brain Heart Infusion (BHI) made by Oxoid and BBL, because it consistently produced 1.63–2.64 log_10 _CFU/ml per hour with all strains and lots tested. BBL-BHI production was low (1.34–1.63 log_10 _CFU/ml per hour) from the beginning and not used further. BHI Oxoid was similar to THB in cell production, but not reproducible with the different lots tested to grow strain INS-E611 (Figure 1). Analysis of these four media by repeated measures ANOVA demonstrated a non-significant difference (P = 0.7976), as expected from the similarity in production during the first few hours of cultivation. However, as illustrated by Figure 2, productivity at the end of each culture (5 hours) was significantly better with supplemented THB compared with standard THB and BHI (P = 0.0022, one way ANOVA). While stationary and death phase with ≤ 8.0 log_10 _CFU/ml was reached before 3 hours with standard broths, supplementation of THB consistently produced ≥ 9.0 log_10 _CFU/ml and prolonged logarithmic growth during Phase 2 up to 4 hours in Tube 6, 5 hours in Tube 7, and 7 hours in Tube 8 (Figure 3). Incubation time during Phase 1 was best at 12 hours, with longer periods resulting in poor growth during Phase 2, mainly determined by media acidification. Adjustment of pH was fundamental during both Phase 1 and Phase 2. For practical reasons, Phase 1 broth was adjusted at the beginning of the 12-hour culture, but Phase 2 broth was adjusted every hour. In Figures 1, 2, 3, the term "supplemented" also implies this protocol for pH adjustment.

### Supplementation and optimization of culture conditions for PRSP

**Figure 4:**
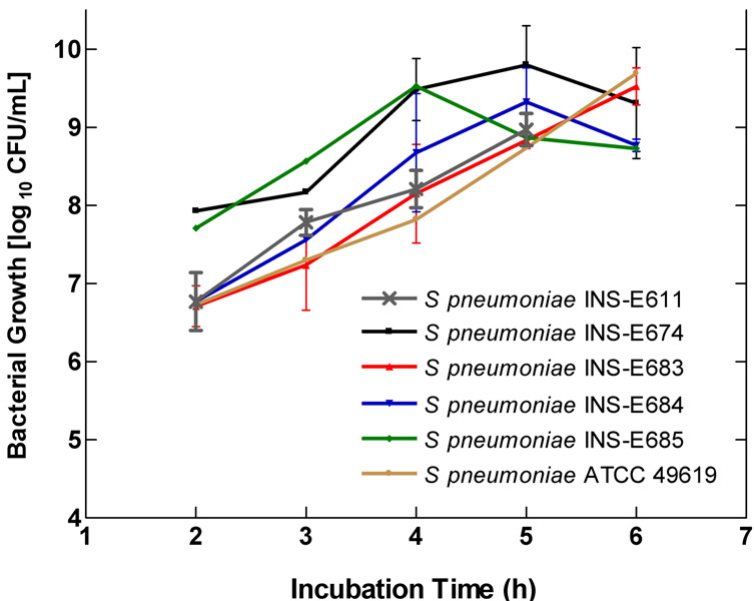
**Growth curves of six strains of PRSP under optimized culture conditions**. The high productivity of viable log-phased bacteria demonstrated for *S. pneumoniae *INS-E611 was reproduced with other 5 strains of PRSP, including diverse serotypes (Table 1). More than 9.0 log_10 _CFU/mL were obtained in a consistent manner with all strains (curves shown correspond to Tube 7, Phase 2). The variability between strains, evidenced by the time to reach maximal growth, is characteristic of *S. pneumoniae*.

Optimized culture conditions produced ≥ 9 log_10 _CFU/ml of log-phased cells in Phase 2. These results were reproducible with all PRSP strains tested (Figure 4) under the following protocol:

**Phase 0: **from frozen stock (skim milk), cells are resuscitated by two consecutive passes on 5% sheep blood TSA supplemented with 0.5% yeast extract and incubated for 15 hours under 5% CO_2 _atmosphere.

**Phase 1: **10 colonies from the second plate are inoculated into 10 ml of supplemented THB (Tube 1), followed by 4 successive 1:10 dilutions into identical liquid media (Tubes 2–5). Supplementation implies pH adjustment to 7.8 with 1N NaOH after addition of 2% yeast extract and 2.5% horse blood. All 5 tubes are incubated for 12 hours under 5% CO_2 _atmosphere, without additional pH adjustment.

**Phase 2: **1 ml of bacterial suspension taken from the most diluted tube with visible growth from Phase 1 is inoculated into 9 ml of supplemented THB (Tube 6) in Phase 2. With most strains and under conditions just described for Phases 0 and 1, Tube 5 or 4 should have visible growth by the end of Phase 1. Tubes 3, 2, and 1 should not be used, because their pH is invariably acidic by the end of Phase 1, usually giving very low cell counts during Phase 2. After inoculation of Tube 6, two successive 1:10 dilutions are made into identical media (Tubes 7 and 8). Each one of these 3 tubes is incubated under 5% CO_2 _atmosphere, and their pH is adjusted every hour to 7.8 with 1N NaOH. We found that optimal incubation periods were 4, 5 and 7 hours for Tubes 6, 7, and 8, respectively.

## Discussion

Our results demonstrate that optimization of culture conditions is an easy, non-expensive and reproducible way to attain penicillin-resistant *S. pneumoniae *growth over 9.5 log_10 _CFU/ml without the need of more complex methods (e.g. chemostat).

The *S. pneumoniae *serotypes used in this study are representative of the most frequent invasive isolates from blood and CSF in Colombia [20,21].

The three standard media used initially (BHI-Ox, BBL-BHI and THB) are recommended by American Society for Microbiology and Clinical Laboratory Standards Institute (CLSI, formerly NCCLS) for *S. pneumoniae *growth, but their productivity was quite different. In contrast with BHI, THB has soy bean peptone and higher pH (7.4 vs 7.8, respectively). Conversely, BBL-BHI has the highest dextrose concentration (3 g/L), which results in marked acidification of the media as anaerobic growth progresses [2]. In this aspect, we confirmed previous findings of acidic pH (under 6.5) inhibiting growth of penicillin resistant and susceptible *S. pneumoniae *strains [12]. To avoid media acidification, we limited the number of colonies produced during Phase 0 to inoculate supplemented THB in Tube 1 (Phase 1) and adjusted pH to 7.8 at the beginning of Phase 1 and every hour during Phase 2. Since pH control is not enough to attain growth over 8.7 log_10 _CFU/ml [12], we addressed two additional characteristics that influence *S. pneumoniae *growth: autolysis and competence. The process of autolysis takes place around the upper part of the logarithmic phase of growth, is started by pheromones liberated by a complex quorum sensor system, and is characterized by extensive cellular death [10,11]. Competence, or the capacity for genetic transformation, is well developed in *S. pneumoniae*, and particularly well in PRSP, but it does not happen without repercussion in bacterial growth, both in vitro and in vivo [3,5,13]. Although the relationship between autolysis and competence not always goes in the same direction, choline mutants (the target of most autolysins) have shown important changes respect to wild type pneumococci, including abnormal growth in long chains instead of diplococci, loss of competence for transformation, resistance to autolysis, and resistance to cell-wall active antibiotics [14]. Based on these facts, we hypothesized that prevention of autolysis could improve production of viable cells even in the logarithmic phase of bacterial growth. Besides offering a suitable culture media, shorter incubation times and separate culture process in three phases allowed generous bacterial growth before triggering autolysis mechanisms. Given that concentrations of 8 log_10 _CFU/ml were obtained 1–2 hours before reaching the maximum incubation periods shown in Figure 3, inoculation of healthy cells in large numbers is possible before triggering autolysis and, probably, while pneumococci are still virulent enough to induce acute pneumonia in animal models.

Goncalves *et al* specifically addressing the optimal growing conditions for *S. pneumoniae*, described a batch cultivation procedure for serotype 23F, suitable for large scale polysaccharide production to manufacture vaccines [2]. They used Hoeprich's medium with different glucose concentrations and supplemented with various amino acids and a vitamin solution, and provided an anaerobic environment with N_2 _or CO_2_. Their results, expressed in terms of biomass and polysaccharide production, are difficult to compare with ours. Moreover, they used autolysis as a way of releasing polysaccharide, whereas we tried to avoid this process to obtain viable bacteria.

This is the first report on culture optimization for maximum growth of PRSP with simple methods, and is directed to facilitate the use of these strains in experimental procedures such as the development of animal models of pneumonia. The cultivation conditions described here generate high concentrations of log-phased bacteria without early and inconvenient induction of autolysis.

## Conclusion

In this study we demonstrate that a standardized inoculum grown in supplemented solid and liquid media with pH adjustment and control of incubation times in three phases produces viable PRSP far beyond the limiting 8 log_10 _CFU/mL. This method should allow improvement in experimental approaches to solve important questions regarding the biology, pathology, and therapeutics of PRSP.

## Methods

### Bacterial strains

Eight clinical strains of *Streptococcus pneumoniae*, obtained from very sick patients nationwide, were supplied by the Colombian Instituto Nacional de Salud (INS). These included six strains non susceptible to penicillin (called further penicillin-resistant): INS-E611, E674 (blood isolates), E676, E678, E683, E684 (cerebrospinal fluid isolates); and two penicillin-susceptible strains: INS-E682 (blood isolate) and E685 (cerebrospinal fluid isolate). A standard strain of PRSP (*S. pneumoniae *ATCC 49619) was used as a control for all experiments (Table 1).

### Susceptibility testing

MIC and MBC to penicillin, ceftriaxone and vancomycin were determined by broth microdilution following CLSI procedures [15]. MIC to the same antibiotics and to chloramphenicol, trimethoprim-sulfamethoxazole and erythromycin had been determined at the Colombian INS using identical methodology.

### Evaluation of baseline culture variables

Strain INS-E611, a penicillin-resistant strain (MIC = 2.0 μg/mL), was selected to test a wide group of variables involved in bacterial growth that could have importance for *S. pneumoniae*. Once the growth dynamics under each variable were determined for this strain, the most productive conditions were standardized and applied to the remaining strains. Bacterial stocks were stored at -70°C in two sets of 100 aliquots per strain. Cryoprotection media included 17% glycerol in trypticase soy broth for one set and skim milk for the other. The process from thawing to the end of variable evaluation was separated in three successive steps that we called phases: Phase 0 for resuscitating the frozen organism on to solid medium (two successive agar plates), Phase 1 for passing it to 10 ml liquid medium a first time (five 16 × 125 glass tubes labeled 1 to 5 with successive 1:10 dilutions), and Phase 2 for a second transfer of log-phased cells into 10 ml fresh liquid medium (three 16 × 125 glass tubes labeled 6 to 8 with successive 1:10 dilutions). The results were determined for all culture variables intervening at each phase and for the whole process by OD_580 nm _(standard broths) and OD_600 nm _(horse-blood supplemented broths) at 20–60 minutes intervals (SPECTRO 22, Labomed, Culver City, CA, USA); quantification of viable CFU per mL was made by dilution plating at least every hour during Phase 2.

These variables were tested by separate and combined at each step: cryoprotection media, addition of 0.5% yeast extract, and incubation time during Phase 0; inoculum size (number of CFU per tube), type of standard culture media, addition of 2% yeast extract and 2.5% horse blood, initial pH adjustment to 7.8 (744 pH Meter, Metrohm Ltd., Switzerland) with 1N NaOH (EK CHEM, Germany), incubation time, and number of the most diluted tube with visible turbidity (used to inoculate Tube 6) during Phase 1; and type of standard culture media, addition of 2% yeast extract and 2.5% horse blood, pH adjustment to 7.8 every hour, incubation time, and bacterial growth rate from Tubes 6, 7 and 8 during Phase 2. All three phases included incubation under 5% CO_2 _atmosphere. Three types of standard culture media were evaluated in Phases 1 and 2: Brain Heart Infusion (BHI-Ox, Oxoid LTD, Basingstoke, Hampshire, UK), BBL™ Brain Heart Infusion (BBL-BHI, Becton Dickinson & Co., Sparks, MD, USA) and Bacto™ Todd Hewitt Broth (THB, Becton Dickinson & Co., Sparks, MD, USA). Horse blood was obtained directly by the research group and defibrinated, lysed, centrifuged and filtered following standard procedures [16]. Sterile quality control agar or broth was set simultaneously with the inoculated media in all culture processes, and incubation temperature was set to 37 and 35°C for experiments with standard and supplemented media, respectively. Experiments were repeated 2–3 times.

### Supplementation and optimization of culture conditions for PRSP

The evaluation of baseline cultures variables allowed the creation of a culture protocol that was tested with all strains using the same phases. The results for the whole process for each strain were standardized by hourly determination of OD_600 nm _and viable CFU/mL count during Phase 2.

### Statistical analysis

Data are presented as means and standard deviations. The significance of differences in productivity of various culture media along time was determined by Repeated Measures ANOVA. For differences in maximal production between these media, one way ANOVA was applied followed by the Bonferroni *t *test. Data were stored, analyzed and graphed with Microsoft Excel v10.2 for Windows (Microsoft Corp., Seattle, WA, USA) and GraphPad Prism v4.0 for Windows (GraphPad Software, San Diego, CA, USA).

## Authors' contributions

AVR carried out the microbiologic experiments, performed the analysis and interpretation of data and drafted the first versions of the manuscript. BES and MA carried out the microbiologic experiments and performed the analysis and interpretation of data. CAR carried out the microbiologic experiments, performed the analysis and interpretation of data and contributed in the study design. AFZ carried out the microbiologic experiments, performed the analysis and interpretation of data and drafted the first versions of the manuscript. OV conceived of the study, directed its design and coordination, obtained funding, directed data analysis, and re-wrote the final version of the manuscript. All authors contributed to the critical revision of the manuscript for important intellectual content and read and approved the final manuscript.

## Role of the Sponsor

The funding organization had no role in the design and conduct of the study; collection, management, analysis, and interpretation of the data; and preparation, review, or approval of the manuscript.

## Figures and Tables

**Table 1:** Serotypes and minimal inhibitory and bactericidal concentrations (MIC / MBC) of six antimicrobial agents against nine strains of *Streptococcus pneumoniae*

**Strains**	**Serotype**	**MIC / MBC [μg/mL]**			MIC* [μg/mL]		
		
		**PEN**	**CRO**	**VAN**	**ERY**	**CHL**	**SXT**
INS-E611	6B	2.00 / 2.00	1.00 / 1.41	0.35 / 0.35	0.03	32.00	4.00
INS-E674	14	2.00 / 2.00	1.00 / 1.00	0.25 / 0.25	0.06	4.00	8.00
INS-E676	14	1.00 / 1.41	1.00 / 1.41	0.35 / 0.70	0.06	4.00	8.00
INS-E678	14	0.12 / 0.12	0.03 / 0.03	0.25 / 0.25	0.06	4.00	16.00
INS-E682^†^	6B	0.01 / 0.01	0.01 / 0.01	0.25 / 0.25	0.06	32.00	0.50
INS-E683	9V	1.00 / 1.00	0.35 / 0.35	0.35 / 0.35	0.06	4.00	32.00
INS-E684	14	1.00 / 1.00	0.50 / 0.50	0.25 / 0.25	0.13	4.00	1.00
INS-E685^†^	1	0.01 / 0.01	0.01 / 0.01	0.25 / 0.25	0.06	2.00	0.50
ATCC 49619	19F	0.46 / 0.46	0.06 / 0.12	0.33 / 0.44	0.06	4.00	0.25

PEN = penicillin; CRO = ceftriaxone; VAN = vancomycin; CHL = chloramphenicol; ERY = erythromycin; SXT = trimethoprim-sulfamethoxazole (only first component values shown)

* MIC determined by Instituto Nacional de Salud (INS), Bogotá, Colombia.

^† ^Breakpoint for penicillin-susceptible strains: MIC ≤ 0.06 μg/mL.

**Table 2:** Comparison of diverse culture variables for productivity of PRSP cells

Variable	Phase 0	Phase 1	Phase 2
Cryoprotection Media	SM > 17%G:TSB	NA	NA
Inoculum (colonies)	NA	10 > 5 or 15 or loopful	NA
Culture Broth	NA	THB > BHI	THB > BHI
Yeast Extract (% YE)	0.5 > 0	2.0 > 0	2.0 > 0
Horse Blood (% HB)	NA	2.5 > 0	2.5 > 0
pH adjustment to 7.8	NA	Initially > NoAdj	q1h > NoAdj
Incubation time (h)	15 > 18 > 24	12 > 15, 18, 24	3*, 5†, 6‡
Ideal dilution (Tube #)	NA	5 > 4 > 1–3	6 = 7 = 8

SM = Skim Milk; > implies "superior to"; NA = non applicable to that Phase; 17%G:TSB = 17% glycerol in trypticase soy broth; THB = Todd Hewitt Broth; BHI = Brain Heart Infusion; NoAdj= no adjustment; *Tube 6; †Tube 7; ‡Tube 8.

**Table 3:** Influence of inoculum size in cell production of *Streptococcus pneumoniae *INS-E611 after 12 hours of Phase 1 culture in supplemented THB

Number of CFU (Phase 0)	OD_600 nm _(Phase 1)	Broth pH (Phase 1)	Phase 1 Production [log_10 _CFU/mL]
1	0.381	7.90	7.278
5	1.878	7.00	8.662
10	0.914 – 1.884	7.08 – 7.32	8.884 ± 0.047
15	1.766	6.28	9.067
Loopful	1.778	6.17	8.851
